# Airway Hyperresponsiveness, but Not Bronchoalveolar Inflammatory Cytokines Profiles, Is Modified at the Subclinical Onset of Severe Equine Asthma

**DOI:** 10.3390/ani13152485

**Published:** 2023-08-01

**Authors:** Thibault Frippiat, Tatiana Art, Irene Tosi

**Affiliations:** 1 Equine Sports Medicine Centre, FARAH Research Centre, Faculty of Veterinary Medicine, University of Liège, 4000 Liege, Belgium; 2 Sportpaardenarts–Equine Sports Medicine, 1250AD Laren, The Netherlands

**Keywords:** horse, equine asthma, methacholine, bronchoprovocation test, airway hyperresponsiveness, bronchoalveolar lavage, inflammatory cytokines, gene expression

## Abstract

**Simple Summary:**

Non-septic airway inflammatory disease in horses has recently been renamed to equine asthma syndrome in reference to human asthma. Airway hyperresponsiveness and inflammation are two closely related components of both human and equine conditions. The understanding of their relationship is crucial for diagnosing, managing, and treating this common disease. One of the shared characteristics of equine and human asthma is the absence of clinical signs in asymptomatic patients. This study aimed to determine the relationship between airway inflammation and hyperresponsiveness at the onset of severe equine asthma. The repeatability of the bronchoprovocation test using methacholine and impulse oscillometry system was first showed in healthy and asymptomatic asthmatic horses. While no clinical or ancillary examination allowed asthmatic horses in clinical remission to be distinguished from healthy ones, a low-dust environmental challenge did not change the results of clinical or conventional ancillary examinations. The methacholine bronchoprovocation test, however, allowed both groups to be distinguished after a 7-day low-dust environmental challenge. The gene expression and the concentrations of pro-inflammatory cytokines showed no change in the bronchoalveolar lavage fluid. No association was found between airway inflammation and hyperresponsiveness, suggesting airway hyperresponsiveness may appear earlier than inflammation at the very early onset of severe equine asthma.

**Abstract:**

Airway hyperresponsiveness (AHR) and inflammation are both observed in human and equine asthma. The aim of this study was to assess the timeline and relationship of both features at the subclinical onset of severe equine asthma (SEA). First, the repeatability of the pulmonary function test (PFT) using impulse oscillometry system, and the methacholine bronchoprovocation test (BPT) were assessed at a 1-day interval on six SEA horses in clinical remission and six control horses. Then, clinical and ancillary tests were performed before and after a 1-week low-dust environmental challenge, including weighted clinical score, respiratory endoscopy, bronchoalveolar fluid cytology, PFT, and BPT. Both PFT and BPT showed acceptable repeatability. No test allowed SEA horses in clinical remission to be distinguished from control, unlike in human patients. Because of the low-dust environment, no significant difference was observed in the results of clinical and conventional ancillary examinations after the challenge. However, SEA horses showed increased AHR after the environmental challenge. At that stage, no signs of inflammation or changes in pro-inflammatory cytokines profiles (quantification and gene expression) were observed, suggesting AHR is present at an earlier stage of equine asthma than airway inflammation. This feature indicates SEA could present in a different disease pathway than neutrophilic human asthma.

## 1. Introduction

For decades, severe equine asthma has been the focus of interest of many specialised equine research centres. First called heaves, it was known as COPD (chronic obstructive pulmonary disease) for its similarities to the homonymous human disorder, and later renamed to RAO (recurrent airway obstruction) to describe its clinical symptomatology. More recently, a group of experts has proposed using the terms “equine asthma syndrome” to characterise the disorder [1], because of the analogies to human asthma in clinical signs, pulmonary remodelling, reversibility of clinical signs and inflammation via eviction of the aeroallergens, and response to therapy. Human asthma is characterised by airway hyperresponsiveness (AHR), inflammation, and narrowing, and different phenotypes have been described in adult patients: early-onset allergic asthma, early-onset allergic moderate-to-severe remodelled asthma, late-onset non-allergic eosinophilic asthma, and late-onset non-allergic non-eosinophilic asthma [2]. Currently, two phenotypes have been proposed within the equine asthma syndrome: mild to moderate (MMEA; formerly called IAD or inflammatory airway disease) and severe equine asthma (SEA; formerly RAO). The latter is a multifactorial and polygenic disease resulting in bronchoconstriction, airway inflammation and hypersensitivity in response to inhaled antigens, in particular the ones present in hay, such as actinomycetes (*Thermoactinomyces vulgari* and *Faeni rectivergula*) and mould spores (*Aspergillus fumigatus*) [3]. The disorder is characterised by a non-septic neutrophilic airway infiltration, mucus hypersecretion, bronchospasm, and airway epithelial hyperplasia [4,5,6]. Susceptible horses exposed to these allergens show an influx of neutrophils into the lungs and a bronchospasm within 5 h to 2 days following exposure [7,8,9,10]. Symptoms mainly include chronic cough, nasal discharge, increased respiratory efforts, and exercise intolerance [11]. Age, obesity, and season (winter and spring) have been showed to be risk factors [12,13]. As observed in humans, clinical signs of SEA are reversible when affected patients are kept away from the antigens [14]. Diagnosis of SEA in clinically affected horses is commonly based on history, clinical signs, and ancillary tests such as endoscopy, cytology of bronchoalveolar lavage fluid (BALf), and pulmonary function tests (PFTs). Unfortunately, most of these tests are not reliable for the diagnosis of SEA in a horse in complete clinical remission (e.g., after a long-term eviction of allergens), nor are bronchoprovocation tests (BPTs) [15,16].

In humans, BPTs have been studied in humans to assess the degree of AHR and in particular for the diagnosis of asthma [17]. Methacholine (acetyl-β-methylcholine) is a parasympathomimetic molecule, similar to acetylcholine, that stimulates the parasympathetic postganglionic muscarinic receptors, thus causing constriction of the bronchial smooth muscles [18]. According to the guidelines of the American Thoracic Society, the principle of the methacholine BPT is to induce a bronchospasm by subjecting patients to inhalations with increasing concentrations of methacholine or histamine, immediately followed by a PFT to monitor changes in breathing resistance. Patients responding to low doses of methacholine are considered hyperreactive, which guides the diagnosis to asthma. Contrariwise, if they respond to higher doses, asthma can be ruled out of the differential diagnoses, even if patients are asymptomatic at the time of examination [19]. The histamine or methacholine BPT has been used in equine research studies to quantify AHR [7,15,16,20,21,22] but is not currently a common tool in clinical veterinary medicine settings.

The underlying pathogenesis of human asthma is multifaceted, involving complex interactions between genetic predisposition, environmental triggers, and dysregulated immune responses within the respiratory system [23]. In recent years, the role of respiratory cytokines has emerged as a key aspect in understanding the intricate immunological mechanisms that drive the pathophysiology of asthma. Respiratory cytokines represent a diverse group of small signalling proteins secreted by immune cells and other cell types within the airways. These cytokines play a pivotal role in orchestrating various immune and inflammatory responses within the respiratory system. In asthma, the dysregulation of these cytokines leads to chronic inflammation, AHR, and remodelling, ultimately contributing to the clinical manifestations of the disease [23]. Among others, tumour necrosis factor alpha (TNF-α) and interleukin-1 beta (IL-1β), two important cytokines mediating inflammation produced by macrophages and epithelial cells, have been associated with the recruitment of neutrophils within airways [24] and have thus been suggested to play a role in human neutrophilic asthma [25,26]. Mice treated with neutralizing anti-IL-1β antibodies showed a markedly reduced response to inhaled antigen [27]. On the other hand, interferon gamma (IFN-γ) is a cytokine with primarily anti-inflammatory properties produced by T lymphocytes. The role and therapeutic potential of IFN-γ in asthma remain controversial [28,29]. Interleukin 8 (IL-8), also known as CXCL8, is a chemokine that plays a crucial role in immune responses and inflammation, thus in pathologic conditions such as asthma. IL-8 is produced by various cell types, including airway epithelial cells, macrophages, and mast cells, in response to different stimuli (e.g., allergens). It functions as a chemoattractant, attracting neutrophils, among others, to the site of inflammation. In asthma, increased levels of IL-8 have been observed in the airways of affected individuals [30]. This elevated production of IL-8 contributes to the recruitment and activation of neutrophils. After hay dust exposure, changes in concentrations and/or gene expression of inflammatory cytokines IL-8, IFN-γ and TNF-α have been observed in the respiratory samples of SEA or neutrophilic MMEA horses [31,32,33,34]. Most of these studies were based on the induction of the disease via a mouldy hay challenge, inducing clinical signs of SEA such as bronchoalveolar neutrophilia.

Understanding the intricate network of respiratory inflammatory cytokines and their specific roles in the pathogenesis of equine asthma is vital for the development of targeted therapeutic interventions. By targeting specific cytokines, it may be possible to modulate the exaggerated immune responses and inflammation seen in asthmatic horses, thereby achieving better disease control and improved quality of life. The aims of the present study were (1) to assess the repeatability of PFTs and methacholine BPTs, and to determine at the subclinical onset of SEA (2) the usefulness of methacholine BPT as early diagnostic tool, and (3) the relationship between AHR and bronchoalveolar inflammatory response. We hypothesised (1) both PFTs and methacholine BPTs would present good repeatability in horses, (2) SEA horses in clinical remission would be impossible to distinguish from healthy ones at the early onset of SEA, and (3) results of methacholine BPTs would be correlated to parameters of airway inflammation.

## 2. Materials and Methods

### 2.1. Horses

A total of 12 horses belonging to the research herd of the Faculty of Veterinary Medicine of the University of Liège were used in this study, including 6 horses with a history of SEA and 6 control horses (CTRL). All procedures were approved by the Animal Ethics Committee of the University of Liege, Belgium (No. 1085) and performed at the Department of Functional Sciences of the Faculty of Veterinary Medicine (agreement No. LA 1610018).

Horses with SEA history were 18.8 ± 3.9 years old (range 12 to 23 years), weighed 513 ± 66 kg (range 430 to 577 kg), and consisted of 4 mares and 2 geldings. Their SEA history status was established via previous observations of a weighted clinical score (WCS) ≥ 15, excessive tracheal mucus production, increased respiratory resistance, and airway inflammation (> 25% neutrophils in BALf) when stabled in stalls and fed mouldy hay. Control horses were 8.0 ± 4.5 years old (range 5 to 17 years), weighed 584 ± 45 kg (range 536 to 636 kg) and included 4 mares and 2 geldings. Their healthy status was established via previous observations of a WCS < 5, no abnormal tracheal mucus production, normal respiratory resistance values, and BALf cytologic assessments (<5% neutrophils, <2% mast cells, <1% eosinophils) when stabled indoor and fed mouldy hay.

All horses were housed in pastures for a minimum of 3 months before the beginning of the protocol, and none of them received any medication during this period. To be included in the study, all horses had to show a WCS of 0, normal haematology and biochemistry, and normal respiratory resistance values.

### 2.2. Experimental Design

#### 2.2.1. Study 1: Within-Subject Repeatability of Respiratory Resistance and Bronchoprovocation Test

**Figure 1:**
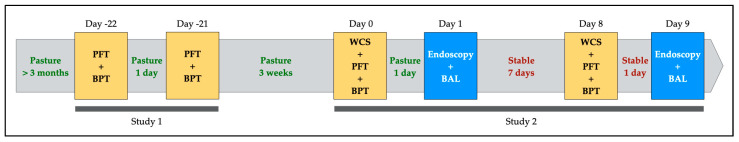
Timeline of the protocol. Study 1 was performed to assess the repeatability of the pulmonary function test (PFT) and bronchoprovocation test (BPT). Study 2 was performed to assess the effect of a mild environmental challenge on airway hyperresponsiveness and inflammation. WCS: weighted clinical score; BAL: bronchoalveolar lavage.

To test the repeatability of PFTs and methacholine BPTs, all horses underwent both examinations on 2 successive days. Between the 2 days, horses were kept in pasture. After the second test, horses were kept in pasture for 3 more weeks before entering the next study. The timeline is shown in Figure 1.

#### 2.2.2. Study 2: Effect of Mild Environmental Challenge on Clinical Score, Respiratory Parameters, Airway Hyperresponsiveness and Bronchoalveolar Inflammation

One day before entering the protocol (Day 0), clinical examination (including WCS), PFTs, and BPTs were performed on all horses. They underwent airway endoscopy and bronchoalveolar lavage (BAL) the day after the BPTs (Day 1), a study having shown that the latter had no effect on BAL cytology after 24 h [35]. Between the two examinations, the horses were kept in pasture. At that point, they were housed for 7 days on low-dust straw in a stable and fed ad libitum low-dust non-mouldy hay and three 0.5 kg meals of concentrates a day. They were allowed to move freely in a sand paddock for 60 min daily. After the 7-day environmental challenge on straw and hay, the same tests as those carried out on Day 0 and 1 were repeated: clinical examination with WCS, PFTs, and BPTs on Day 8 and airway endoscopy and BAL on Day 9. Between the two last days, the horses were kept indoors. The timeline is shown in Figure 1.

### 2.3. Clinical Procedures

#### 2.3.1. Weighted Clinical Score (WCS)

Clinical examination was used to establish a WCS ranging from 0 to 23, based on respiratory rate, nasal discharge, abdominal expiratory lift, nasal flaring, tracheal sounds, bronchial tones, presence of crackles and wheezes, and cough reflex (Table 1) [36]. A higher score indicated more severe clinical manifestations of the disease and an increased respiratory resistance during disease exacerbations [37,38]. Horses with a score between 5 and 10 were classified as mildly affected, those with a score between 11 and 14 as moderately affected, and horses with a score ≥ 15 as severely affected.

#### 2.3.2. Airway Endoscopy and Bronchoalveolar Lavage Fluid (BALf) Collection

Horses were sedated using romifidine hydrochloride (0.04 mg/kg IV) and butorphanol tartrate (0.04 mg/kg IV) and restrained using a nose twitch. Airway endoscopy was conducted using a flexible sterilised video-endoscope (length 2.6 m, external diameter 10.5 mm). Tracheal mucus quantity score [39] and tracheal septum thickness score [40] were assessed.

The endoscope was directed down into the right lung until its tip was wedged against the walls of a peripheral bronchus [41], and the BAL was performed via the biopsy channel of the endoscope. A first bolus of 100 mL of prewarmed (37 °C) sterile saline was introduced and gently aspirated. Three additional 50 mL syringes of saline were then introduced and collected one by one. The first sample (first bolus of 100 mL) was centrifuged at 2000 rotations per minute (rpm) for 8 min. Aliquots of 500 μL were prepared from the supernatant thereof and frozen at −80 °C until the subsequent assay of cytokines. The second sample (an additional 150 mL), pooled with the rest of the first, was used for BALf cytology and measurement of the cytokine gene expression in the leukocytes.

#### 2.3.3. Pulmonary Function Test (PFT)

The pulmonary function test was performed using an impulse oscillometry system (IOS; IOS MasterScreen, Jaeger GmbH, Würzburg, Germany), a forced oscillation technique measuring respiratory resistance and reactance from 3 to 10 Hz during spontaneous breathing [42]. Briefly, multifrequency pressure impulses produced by a loudspeaker are transmitted to the horse’s respiratory system via a flexible tube connected to an airtight mask placed on the horse’s head. The measuring component of the IOS, placed between the flexible tube and facemask, contains a heated pneumotachograph connected to pressure transducers. Pressure and airflow signals measured in the time domain are processed via Fast Fourier Transformation to obtain respiratory resistance and reactance in a range of frequencies from 3 to 10 Hz. In the present study, only measures of respiratory resistance values obtained at 3 Hz (R_3_) were recorded, as a previous study showed that low frequencies are more sensitive than high frequencies during methacholine BPTs [43]. The total duration of a single test was 30 s, and the resulting data were the average of the recorded respiratory cycles. The test was repeated 3 times, and the mean of the 3 measures was calculated and used further as R_3_. Prior to each experiment, the flow volume of the system was calibrated by use of a 2-L calibration pump (Medisoft, Dinant, Belgium).

#### 2.3.4. Methacholine Bronchoprovocation Test (BPT)

**Figure 2:**
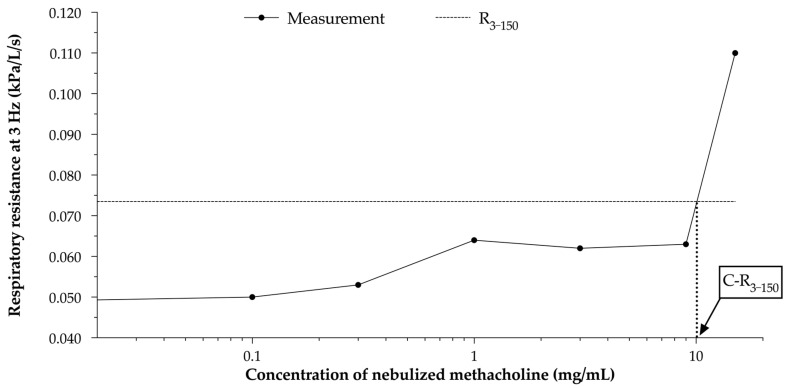
Example of calculation of the concentration of methacholine needed to reach the threshold in a control horse during a methacholine bronchoprovocation test. R_3–150_: threshold value of bronchial reactivity, corresponding to 150% of the value of the baseline respiratory resistance at 3 Hz; C-R_3–150_: concentration of nebulised methacholine when respiratory resistance reached R_3–150_.

Based on the measured R_3_, the threshold of respiratory resistance (R_3–150_) was calculated, corresponding to 150% of the baseline value [44]. An equine nebulization mask was fitted, sealed tightly, and connected to a compressor nebuliser for medical aerosols (HorseNeb, Soinvett, Thuin, Belgium). Following this, BPTs were performed by nebulizing 1 mL saline with methacholine chloride at increasing concentrations (0, 0.1, 0.3, 1, 3, 9, and 15 mg/mL, respectively). Nebulised solutions were administered over 2 min, after which the mask was retrieved. After 2 more minutes without the mask, a PFT was performed. The methacholine BPT was stopped when more than 2 coughs occurred in close succession, in case of acute respiratory distress, or when R_3_ reached R_3–150_. The concentration of methacholine needed to obtain R_3–150_ (C-R_3–150_) was then calculated (Figure 2).

### 2.4. Laboratory Procedures

#### 2.4.1. Bronchoalveolar Inflammatory Cytokines Quantification via Enzyme-Linked Immunosorbent Assay (ELISA)

The concentrations of IFN-γ, IL-1β, IL-8 and TNF-α were measured via ELISA homologs tests for equine species (IL-1β/INF-γ: Kingfisher Biotech, St. Paul, MN, USA; IL-8/TNF-α: antibodies-online GmbH, Aachen, Germany). The plates were already coated with antibodies when delivered. The procedure was performed according to the manufacturers’ recommendations in duplicates. The absorbance of each well was measured via a plate reader at a wavelength of 450 nm. Analyte concentrations for each horse and sampling was normalised to BALf volume recovered from the first 100 mL of instilled saline.

#### 2.4.2. BALf Cytology

The BALf was maintained at 4 °C in EDTA tubes until further processing within 1 h of collection. Preparation of the cells was performed by spreading 250 μL of BALf on a microscope slide via centrifugation (Cytospin, Shandon, Pittsburgh, PA, USA) at 1000 rpm for 3 min, then dried and stained in accordance with the May–Grünwald–Giemsa method. Differential cell counts were performed by 2 blinded operators counting a minimum of 300 cells, excluding epithelial cells and erythrocytes, and the results of the 2 readings were averaged. A cell count was also carried out via a Thoma cell counting chamber (Marienfeld, Lauda-Königshofen, Germany).

#### 2.4.3. Measurement of Gene Expression of Leukocytes via Real-Time Quantitative Polymerase Chain Reactions (RT-qPCR)

The remainder of the samples were centrifuged (1000 rpm for 3 min) in order to recover the pellet of cells. The extraction of RNA was performed according to the manufacturer’s recommendations (Trizol Reagent, Thermo Fisher Scientific Inc., Epsom, UK), and samples were frozen at –80 °C until further analysis. Quality and quantity control of total RNA was performed using a spectrophotometer (Nanodrop ND-1000, Isogen Life Science, Utrecht, the Netherlands). The 260:280 nm absorbance ratio was considered good when greater than 1.8. Quality of RNA was also determined via agarose gel electrophoresis. Reverse transcription was performed on the RNA to obtain cDNA, according to the recommendations of the kit (RevertAid H Minus, First Strand cDNA Synthesis Kit, Fermentas, Belgium). Five reference genes—GAPDH, HPRT, RPL32, SDHA and TFRC—were tested to assess their stability using the software program qbasePLUS 2.0 (Biogazelle, Zwijnaarde, Belgium). The genes of interest were those coding for the cytokines tested during the ELISA tests, namely IFN-γ, IL-1β, IL-8, and TNF-α (Table 2). RT-qPCR was performed with ABSolute Blue QPCR SYBR Green (Thermo Fisher Scientific Inc., Epsom, UK) on a thermocycler for RT-qPCR (Applied Biosystems, Carlsbad, CA, USA).

### 2.5. Statistical Analysis

Analyses were performed using Prism 9.5.1 (GraphPad Software, San Diego, CA, USA). All analyses were considered significant when *p* < 0.05. Results are shown as mean ± standard deviation (SD) or median [interquartile range (IQR)] for normally or non-normally distributed data, respectively, according to a Shapiro–Wilk test.

#### 2.5.1. Study 1: Within-Subject Repeatability of Respiratory Resistance and BPT

To test the repeatability of PFTs and methacholine BPTs, agreement between measurements on 2 consecutive days was quantified by estimating the 95% limits of agreement (LoA), as proposed by Bland and Altman [46]. Pearson’s or Spearman’s rank correlation coefficients and their 95% confidence intervals (CI) were calculated for normally or non-normally distributed data, respectively, to assess the relationship between both measurements on either of the 2 examinations.

#### 2.5.2. Study 2: Effect of Mild Environmental Challenge on Clinical Score, Respiratory Parameters, Airway Hyperresponsiveness and Alveolar Inflammation

A Kruskal–Wallis test with Dunn’s multiple comparisons correction was performed to assess the significant difference in all variables between measurements (Day 1 versus Day 8 or Day 2 versus Day 9) and between groups (SEA versus CTRL). Cohen’s *d* was applied to assess the effect size as small (<0.20), medium (0.20 to 0.50), or large (>0.50) on the significant differences between groups and/or time points. All data were used to assess the correlation between AHR and each parameter of inflammation via a nonparametric Spearman correlation coefficient.

## 3. Results

All procedures were well tolerated by all horses. No administration of bronchodilator was required to treat a severe bronchospasm after the methacholine BPTs.

### 3.1. Within-Subject Repeatability of Respiratory Resistance and BPT

**Figure 3:**
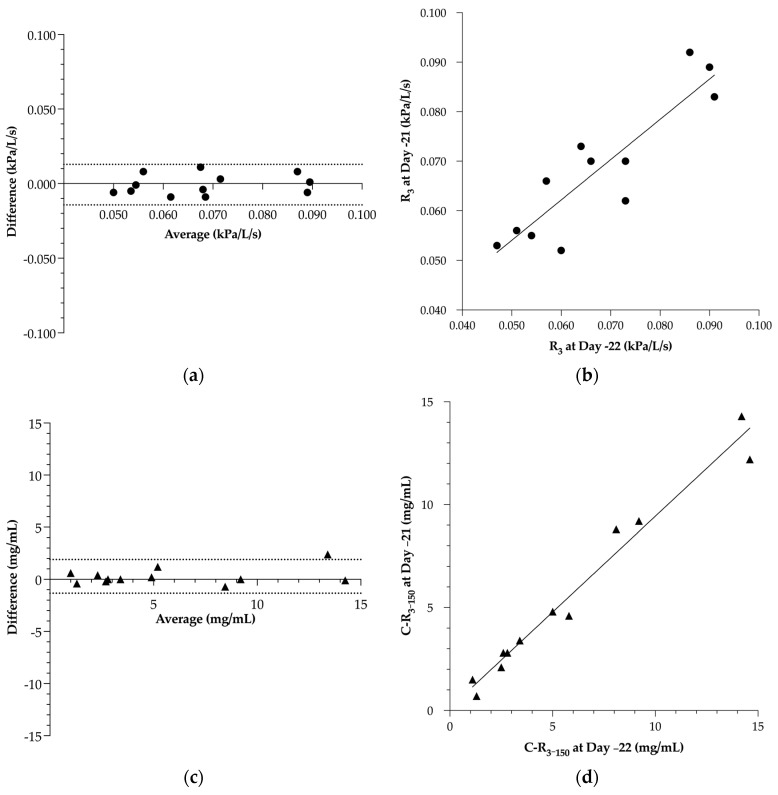
Assessment of the repeatability of the pulmonary function test (**a**,**b**) and methacholine bronchoprovocation test (**c**,**d**) on 2 successive days in 12 horses (6 severe equine asthma-affected horses and 6 control horses). The agreement was quantified via the Bland–Altman method based on (**a**) respiratory resistance at 3 Hz (R_3_), and (**c**) concentration of methacholine needed to obtain the threshold corresponding to 150% of R_3_ (C-R_3–150_). Correlation between the 2 measurements was assessed in (**b**) R_3_ via a Pearson correlation coefficient, and in (**d**) C-R_3–150_ via a Spearman’s rank correlation coefficient.

For the PFTs, the Bland–Altman agreement of R_3_ showed a bias of −0.001 ± 0.007 kPa/L/s, with an LoA ranging from −0.014 to 0.013 kPa/L/s (Figure 3a). This ancillary examination showed a significant Pearson’s correlation coefficient of 0.98 with a 95% CI of 0.92 to 0.99 (Figure 3b). The regression line showed a slope of 0.81 ± 0.13 with an intercept at 0.013 ± 0.009 kPa/L/s.

For the methacholine BPTs, the agreement on C-R_3–150_ showed a bias of −0.28 ± 0.83 mg/mL, with an LoA ranging from −1.34 to 1.90 mg/mL (Figure 3c). The ancillary examination showed a significant Spearman’s rank correlation coefficient of 0.89 with a 95% CI of 0.64 to 0.97 (Figure 3d). The regression line showed a slope of 0.93 ± 0.05 with an intercept at 0.12 ± 0.38 mg/mL.

### 3.2. Effect of Mild Environmental Challenge on Clinical Score, Respiratory Parameters, Airway Hyperresponsiveness and Bronchoalveolar Inflammation

**Figure 4:**
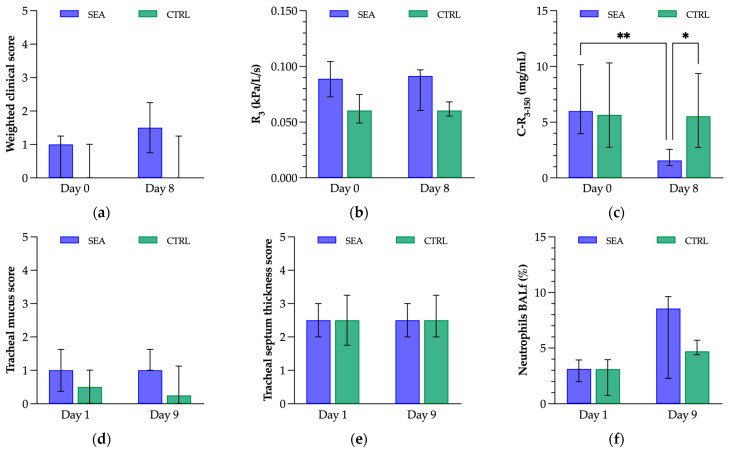
Results of the clinical and ancillary examinations in 6 severe equine asthma (SEA) and 6 control (CTRL) horses before (Day 0 or 1) and after (Day 8 or 9) a mild environmental challenge. (**a**) Weighted clinical score; (**b**) respiratory resistance at 3 Hz (R_3_); (**c**) results of the methacholine bronchoprovocation test (C-R_3–150_: concentration of methacholine needed to obtain the threshold corresponding to 150% of R_3_); (**d**) tracheal mucus score; (**e**) tracheal septum thickness score; (**f**) neutrophils in the bronchoalveolar lavage fluid (BALf). Results are shown as median ± interquartile range. Asterisks indicate a significant difference between measurements, where ** *p* < 0.01 and * *p* < 0.05.

At the beginning of the protocol, SEA horses could not be distinguished from CTRL horses by means of clinical and ancillary examinations. Exposure to indoor environment for 7 days had no significant effect on WCS, PFT, or endoscopic findings in either group (Figure 4). A non-significant increase in BAL neutrophilia was observed in both groups but mainly in SEA horses. Contrariwise, the only significant observation was a decrease in C-R_3–150_ in SEA horses after compared with before the environmental challenge, while C-R_3–150_ did not change in CTRL horses after compared with before the environmental challenge. The decrease in C-R_3–150_ in SEA horses allowed us to significantly distinguish SEA from CTRL horses based on C-R_3–150_ (SEA: 1.68 ± 0.87 mg/mL; CTRL: 4.99 ± 2.56 ng/mL) after the environmental challenge. Both observations showed a large effect size.

The median of the BAL neutrophilia after environmental challenge was 8.5% in SEA horses, demonstrating that the performed challenge was of mild intensity and/or limited duration. The total number of cells in the BALf was not significantly modified (24.2 ± 6.5 × 10^3^ cells/mL on Day 1 versus 22.0 ± 2.6 × 10^3^ cells/mL on Day 9).

**Figure 5:**
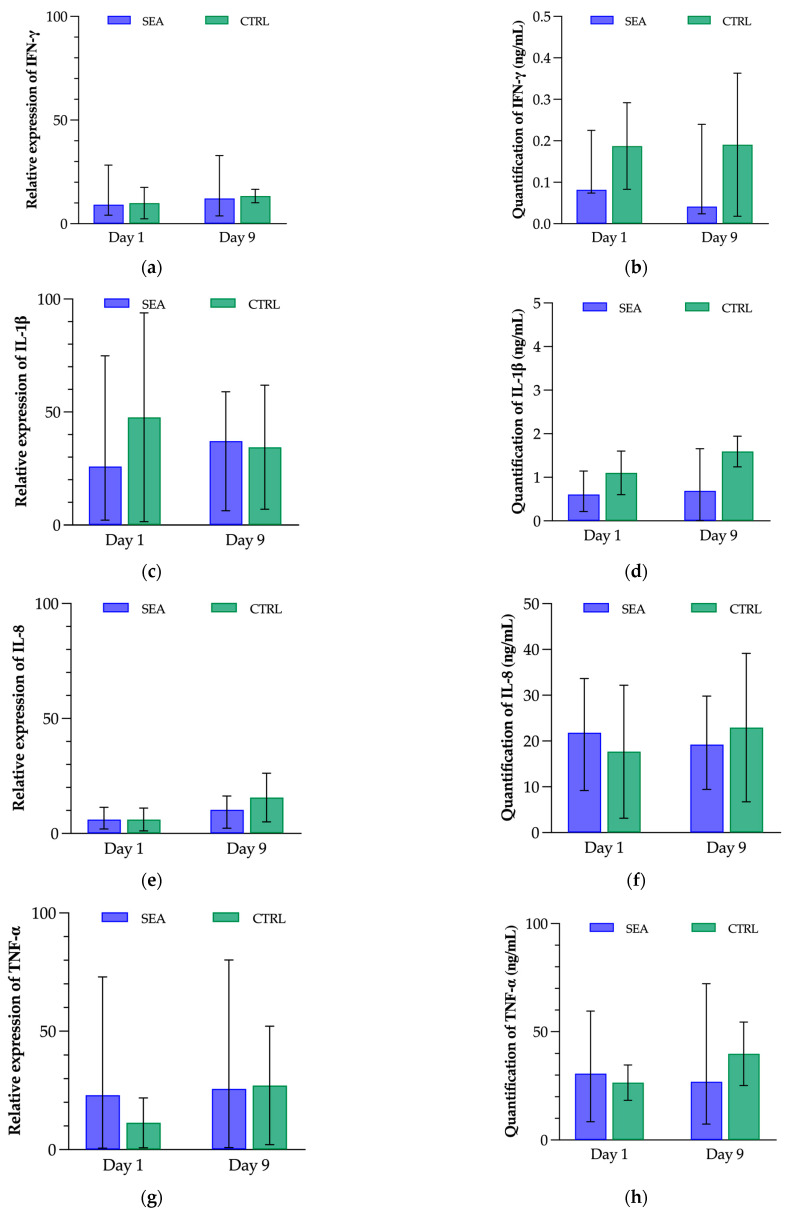
Profiles of interferon gamma (IFN-γ), interleukin-1 beta (IL-1β), interleukin-8 (IL-8), and tumour necrosis factor alpha (TNF-α) in 6 severe equine asthma-affected horses (SEA) and 6 control horses (CTRL) before (Day 1) and after (Day 9) a mild environmental challenge. (**a**,**c**,**e**,**g**) Relative gene expression of (**a**) IFN-γ, (**c**) IL-1β, (**e**) IL-8, and (**g**) TNF-α, compared to reference genes GAPDH, RPL32, SDHA, and TFRC. (**b**,**d**,**f**,**h**) Concentrations of (**b**) IFN-γ, (**d**) IL-1β, (**f**) IL-8, and (**h**) TNF-α in the bronchoalveolar lavage fluid. No significant difference was observed between groups of horses, or before versus after environmental challenge.

Among the five reference genes, GAPDH, RPL32, SDHA, and TFRC were identified as the most stable and were therefore used for the measurement of cytokine gene expression. Results were indicated as relative expressions, related to the median per group. No significant variation in gene expression for IFN-γ, IL-1β, IL-8, or TNF-α was observed. The results of the quantification of these cytokines in the BALf did not show any significant difference between groups nor between time-points (Figure 5).

When analysing all data from both groups at both examination times, a correlation between AHR and WCS, BALf neutrophilia, and tracheal mucus score was observed but not between AHR and respiratory resistance, tracheal septum thickness score, or any of the cytokine concentrations or gene expressions (Table 3).

## 4. Discussion

Airway hyperresponsiveness refers to an exaggerated bronchoconstrictive response to various stimuli, leading to airway narrowing. It is a hallmark characteristic of asthma-like conditions in humans. In horses, AHR can be triggered by environmental factors such as dust, allergens, or moulds [3]. Modified AHR is typically assessed in horses via measurements of lung function using techniques such as PFTs or BPTs [42,47].

The present study aimed to assess the repeatability of PFTs and methacholine BPTs, determining the usefulness of methacholine BPTs as an early diagnostic tool at the subclinical onset of SEA, and observing the relationship between AHR and bronchoalveolar inflammatory response. The study design included two parts: a repeatability study including PFTs and methacholine BPTs and a study on the effect of an environmental challenge on respiratory parameters and inflammatory cytokines.

The first finding of our study was the good repeatability of PFTs using IOS and of methacholine BPTs in both SEA horses in clinical remission and control horses. Previous studies showed the repeatability and/or reproducibility of these tests in horses [47,48]. However, these studies were not performed in horses with a history of SEA and were based on different techniques than those performed in the present study. Nolen-Watson et al. showed an acceptable reproducibility of histamine BPTs after short (one to four weeks) and long (three to twelve months) intervals using flowmetric plethysmography on 29 healthy horses [47]. In a cross-over design on 19 MMEA horses, flowmetric plethysmography diagnosed AHR more frequently than did forced oscillatory mechanics, another technique for PFT [48]. In our study, we tested the repeatability of BPT using IOS as previously described [43,44]. Next to an acceptable intra-individual agreement, the methacholine BPT presented a good feasibility.

Results of clinical and ancillary examinations did not allow us to discriminate between SEA horses in clinical remission. In these horses, WCS, R_3_, endoscopic findings (tracheal mucus score and tracheal septum thickness score), and bronchoalveolar inflammation were not different than in CTRL horses, confirming previous observations in other studies [49,50]. In human medicine, BPTs allow for the diagnosis of asthma, even in patients in clinical remission [51,52]. We showed that this feature is not applicable to horses, as SEA horses in clinical remission showed no significantly different AHR than CTRL horses, as previously shown [15,16].

When stabled for one week, results of clinical and ancillary examinations still did not allow us to distinguish SEA from CTRL horses. This finding is most probably due to the low severity of the environmental challenge by use of low-dust straw and low-dust not-mouldy hay. The reason of this choice was that we aimed to assess if ancillary examinations, and in particular the methacholine BPT, could detect changes in respiratory parameters at the early stages of SEA. A high-dust environmental challenge induced significant changes in clinical and respiratory parameters in SEA horses [4,8,39,40,49,53,54]. In the present study, SEA horses were qualified as such via clinical and ancillary examinations in previous studies. It included a BALf neutrophilia above 25% as previously defined by an international consensus on equine asthma [1]. However, in the present study, SEA horses showed an average BALf neutrophilia below 10% after one week of indoor stabling. We attributed this lower percentage of neutrophils than expected to the low-dust environment as described above.

Nevertheless, the results of the methacholine BPTs did change significantly in SEA horses after the seven-day environmental challenge but not in CTRL horses. Despite the absence of increased BALf neutrophilia, SEA horses showed an increased AHR after one week in a low-dust environment, as shown by a significant decrease of the concentration of methacholine needed to induce an increase of 50% of the respiratory resistance at 3 Hz. This finding confirmed previous observations of increased AHR in SEA horses when exposed to allergens [22,54,55,56]. This increased AHR seems to persist for at least 3 days to 2 weeks after allergens removal [16,56].

Airway hyperresponsiveness and airway inflammation are two closely related components of respiratory disease in horses. The understanding of their relationship is crucial for the diagnosis, management, and treatment of respiratory conditions commonly seen in horses, such as equine asthma. In our study, cytokines quantification and gene expression were not significantly affected before versus after environmental challenge on both SEA and CTRL horses. A previous study on 33 healthy horses showed no direct association between cytological evidence of airway inflammation and AHR [57]. Airway reactivity and BALf cytology showed no concordance in 45 other horses presented for unexplained poor performance and/or chronic cough [58]. Authors concluded that omitting BPT could lead to the underdiagnosis of MMEA in racehorses presenting clinical signs suggestive of MMEA. Both studies were conducted using histamine and, to our knowledge, no studies showing a relationship between airway inflammation and AHR have been conducted yet in SEA horses. Lavoie et al. showed, however, an increased mRNA expression of IFN-γ, interleukin-4, and TNF-α in MMEA horses compared to control horses, while the expression of IL-1β and IL-8 was not affected in horses with SEA [45]. The expression of IL-1β was, interestingly, increased with airway neutrophilia. Richard et al. reported higher concentrations of IFN-γ and TNF-α in the BALf of MMEA horses; the latter was observed in particular in neutrophilic subtype of MMEA [59]. Another study showed no change in gene expression for IFN-γ, IL-1β, and IL-8 after a 1-day environmental challenge [32].

The absence of changes in the concentration of the selected cytokines in our study could suggest that these cytokines are not directly involved in the onset of AHR. Similar observations have also been reported in humans [60,61]. Other reasons for an increased AHR have been proposed, such as the lower quality of the bronchial epithelial fluid, a modified permeability of the bronchial epithelium allowing enhanced fixation of methacholine with the membrane receptors of smooth muscle fibres, an increased excitability of receptors, or a modification of the basic tone of smooth muscle fibres [62,63,64].

The present study presents three main limitations. The first one is the relatively small cohort of horses studied. The effect size on the significant results was however determined as large by its Cohen’s *d*. A second limitation is the relatively low changes in respiratory parameters due to the low severity of the environmental challenge as previously described. Medical history and clinical features of SEA and CTRL horses were well known by the researchers as they had been followed over a long period of time, allowing the inclusion in either group. Finally, we have limited the analyses of cytokines to IFN-γ, IL-1β, IL-8, and TNF-α for technical reasons. Other cytokines may be involved, potentially implicated in a predominant T helper type 2 (Th2) and Th17 driven response, as suggested elsewhere [65].

## 5. Conclusions

The present study confirms that PFTs using IOS and BPTs using methacholine present a good repeatability in horses. Furthermore, human and equine asthma differ in the sense that equine asthmatic patients in clinical remission do not show increased AHR compared to healthy ones. While human asthmatic patients in clinical remission can be diagnosed by use of methacholine BPT, this observation does not apply to SEA horses when in clinical remission.

None of the studied inflammatory parameters, gene expression, or concentration of pro-inflammatory cytokines were modified by the low-dust environmental challenge, while AHR increased in SEA horses. These findings suggest in this small cohort of horses that AHR is appearing earlier than airway inflammation at the subclinical onset of SEA.

## Figures and Tables

**Table 1:** Clinical parameters were used to assess the weighted clinical score (range 0 to 23) of the horses (based on [36]).

Descriptor	Observation	Score
**Respiratory rate** **(breaths/min)**	<16	0
	16–20	1
	21–25	2
	26–30	3
	>30	4
**Nasal discharge**	None	0
	Serious	1
	Mucous	2
	Mucopurulent	3
**Nasal flaring**	None	0
	Present	1
**Abdominal lift**	None	0
	Mild movement of abdomen and/or thorax and/or anus	1
	Pronounced movement of abdomen and/or thorax and/or anus	3
**Tracheal sounds**	Normal (tubular sound)	0
	Increase in intensity	1
	Mucus movement	3
**Bronchial tones**	Normal	0
	Abnormal	2
**Crackles**	None	0
	Present	2
**Wheezes**	None	0
	Present	2
**Cough**	None	0
	Inducible by moderate pressure signal on larynx	1
	Intermittent	2
	Paroxysmal	3

**Table 2:** Pairs of primers used for real-time quantitative polymerase chain reactions. IFN-γ: gamma interferon; IL-1β: interleukin-1 beta; IL-8: interleukin-8; TNF-α: tumour necrosis factor alpha. ^1^ Obtained from [45]. ^2^ Obtained from http://genome.ucsc.edu (accessed on 18 August 2011).

Cytokine	Sense (5′ → 3′)	Antisense (3′ → 5′)
IFN-γ ^1^	CTT GTG CCT CAG CCT CTT CTC CTT C	GCG CTG GAC CTT CAG ATC AT
IL-1β ^2^	CTT CCA AGA CCT GGA CCT CA	GCC ACA ATG ATT GAC ACG AC
IL-8 ^1^	CTT TCT GCA GCT CTG TGT GAA G	GCA GAC CTC AGC TCC GTT GAC
TNF-α ^2^	AGC CTC TTC TCC TTC CTC CTT	CAG AGG GTT GAT TGA CTG GAA

**Table 3:** Spearman’s rank correlation coefficient calculated between the airway hyperresponsiveness (AHR) and clinical respiratory resistance or inflammatory parameters in 12 horses (6 severe equine asthma-affected horses and 6 control horses). The AHR was determined via a methacholine bronchoprovocation test. WCS: weighted clinical score; R_3_: respiratory resistance at 3 Hz; BALf: bronchoalveolar lavage fluid; IFN-γ: gamma interferon; IL-1β: interleukin-1 beta; IL-8: interleukin-8; TNF-α: tumour necrosis factor alpha. Asterisks indicate a significant correlation where * *p* < 0.05.

Parameter		r (95% CI)	*p*-Value
WCS		−0.49 (−0.75–−0.10)	0.01 *
R_3_		0.18 (−0.25–0.56)	0.39
Tracheal mucus score		−0.48 (−0.72–−0.02)	0.04 *
Tracheal septum thickness score		0.40 (−0.02–0.70)	0.05
BALf neutrophilia		−0.42 (−0.71–0.00)	0.04 *
IFN-γ	Quantification	0.24 (−0.30–0.67)	0.36
	Gene expression	−0.07 (−0.56–0.45)	0.79
IL-1β	Quantification	−0.21 (−0.34–0.65)	0.44
	Gene expression	0.16 (−0.38–0.62)	0.56
IL-8	Quantification	0.10 (−0.43–0.58)	0.70
	Gene expression	−0.09 (−0.57–0.44)	0.74
TNF-α	Quantification	−0.06 (−0.54–0.63)	0.85
	Gene expression	−0.00 (−0.51–0.51)	0.99

## Data Availability

The data presented in this study are available upon request from the corresponding author.
